# Comparative transcriptomic and metabolomic analyses reveal differences in flavonoid biosynthesis between PCNA and PCA persimmon fruit

**DOI:** 10.3389/fpls.2023.1130047

**Published:** 2023-02-27

**Authors:** Yiru Wang, Yujing Suo, Weijuan Han, Huawei Li, Zhenxu Wang, Songfeng Diao, Peng Sun, Jianmin Fu

**Affiliations:** ^1^ Research Institute of Non-Timber Forestry, Chinese Academy of Forestry, Zhengzhou, China; ^2^ Food Inspection Center, Henan Institute of Product Quality Technology, Zhengzhou, China

**Keywords:** persimmon, fruit, flavonoid biosynthesis, metabonomic, transcriptomic

## Abstract

The fruit of the persimmon (*Diospyros kaki*.) has high economic and nutritional value and is rich in flavonoids. Flavonoids are essential secondary metabolisms in plants. The association between persimmon astringency and changes in the proanthocyanidins (a flavonoid subclass) content is well-known. However, information on the relationships between different astringency types and other flavonoid subclasses and biosynthetic genes is more limited. In this study, an initial correlation analysis between total flavonoids and fruit astringency type, and KEGG analysis of metabolites showed that flavonoid-related pathways were linked to differences between mature pollination-constant non-astringent (PCNA) varieties (‘Jiro’ and ‘Yohou’) and pollination-constant astringent (PCA) fruit varieties (‘Zhongshi5’ and ‘Huojing’). Based on these findings, variations in the expression of genes and metabolites associated with flavonoid biosynthesis were investigated between typical PCNA (‘Jiro’) and PCA (‘Huojing’) persimmons during fruit development. The flavonoid concentration in ‘Huojing’ fruit was significantly higher than that of ‘Jiro’ fruit, especially, in levels of proanthocyanin precursor epicatechin and anthocyanin cyanidin derivatives. Combined WGCNA and KEGG analyses showed that genes such as *PAL*, *C4H*, *CHI*, *CHS*, *F3H*, *F3’5’H*, *FLS*, *DFR*, *ANR*, *ANS*, and *UF3GT* in the phenylpropanoid and flavonoid biosynthesis pathways may be significant factors impacting the proanthocyanin precursor and anthocyanin contents. Moreover, interactions between the *R2R3MYB* (*evm.TU.contig7272.598*) and *WD40* (*evm.TU.contig3208.5*) transcription factors were found to be associated with the above structural genes. These findings provide essential information on flavonoid biosynthesis and its regulation in the persimmon and lay a foundation for further investigation into how astringency types affect flavor components in PCNA and PCA persimmons.

## 1 Introduction

Flavonoids are essential secondary metabolites in plants and include more than 10 000 structural variants (Quideau et al., 2011; Mikhailova et al., 2022; Tahmaz and Soylemezoglu, 2022). Flavonoids are divided into six subclasses according to substitutions and B-ring attachments to the basic skeletal structure, namely, flavones, flavonols, anthocyanins, flavanols, flavanones, and isoflavones (Winkel-Shirley, 2001; Sasaki and Nakayama, 2015). These compounds have essential physiological and ecological functions in regulating plant growth and development, flower coloring, fruit flavor, physiological activities, and adaptation to abiotic stress in plants (Havsteen, 2002; Tohge et al., 2018). Flavonoids produced by plants have many health benefits for humans, including antibacterial, antiparasitic, anti-inflammatory, anticancer, and anti-aging properties (Dias et al., 2021). Flavonoids have various pharmaceutical activities and often act as antioxidants according to their free radical-scavenging abilities (Kumar and Pandey, 2013). Thus, flavonoids have gained increasing attention and are widely used in the food, cosmetic, and pharmaceutical industries.

The flavonoid synthesis pathway is relatively well understood in model plants (Routaboul et al., 2006; Tohge et al., 2017). The structural genes encoding enzymes in the pathway have been identified, including genes encoding phenylalanine lyase (*PAL*), anthocyanidin synthase (*ANS*), cinnamic acid hydroxylase (*C4H*), coumadin CoA ligase (*4CL*), chalcone synthase (*CHS*), flavonoid 3’-hydroxylase (*F3’H*), chalcone isomerase (*CHI*), flavonol synthase (*FLS*), flavonoid 3’5’-hydroxylase (*F3’5’H*), and the other key genes (Routaboul et al., 2006; Saito et al., 2013; Chen and Li, 2016). Studies on flavonoid biosynthesis have been conducted in many horticultural plants such as *Vitis vinifera* (Azuma et al., 2012), *Malus×domestica* (Henry-Kirk et al., 2012), *Ziziphus jujuba* (Zhang et al., 2020), and others. Furthermore, flavonoid biosynthesis is known to be influenced by the environment, developmental stage, plant variety, temperature, and tissue type (Azuma et al., 2012; Wen et al., 2020). Transcription factors (TFs) involved in the flavonoid biosynthesis pathway have also been identified, such as *R2R3MYB*, *bZIP*, *WD40*, and *bHLH* (Chen et al., 2022).

Persimmon (*Diospyros kaki* Thunb.) is a fruit tree that belongs to the family Ebenaceae and has a long history of cultivation (Saleem et al., 2022). As a major fruit variety with a unique flavor, the persimmon has become increasingly popular and has high commercial value in Asian countries (Han et al., 2022). Flavonoids produced by plants have many health benefits for humans and play crucial roles in both the fruit quality and its economic value (Dias et al., 2021; Xie et al., 2022). Persimmons are rich in phytochemicals such as flavonoids, carotenoids, triterpenoids, fatty acids, and vitamin C (Direito et al., 2021), with flavonoids being the main active antioxidants (Sun et al., 2011). The persimmon fruit has a variety of pharmacological poroperties, including antiadipogenic, hypocholesterolemic, antioxidant, anti-inflammatory, and antitumor poroperties, due to its flavonoid components such as hesperidin, naringin, and nobiletin (Direito et al., 2021). Besides, flavonoids such as proanthocyanidins are associated with the astringency and flavor of the persimmon fruits (Zheng et al., 2021).

Proanthocyanidins (PAs), also known as condensed tannins, are a subclass of flavonoids and consist of oligomers of catechins that are biosynthesized through the flavonoid branch of the phenylpropanoid pathway (Dixon et al., 2005; Zheng et al., 2021). High concentrations of insoluble PAs usually lead to astringency in persimmon fruit. Based on the fruit characteristics, persimmons can be classified into four astringency types, namely, the pollination-variant non-astringent (PVNA), PCNA, PCA, and pollination-variant astringent (PVA) types (Yonemori et al., 2000). In China, almost all persimmon cultivars belong to the PCA type, and no PVA and PVNA types are found (Du et al., 2009). The quality and flavor of the persimmon fruit vary greatly, with significant differences between PCNA and PCA persimmons. The fruit astringency type is not only affected the proanthocyanidins content but also the accumulation of total soluble solids, individual sugars, total phenolics, and total flavonoids (Novillo et al., 2016; Yildiz and Kaplankiran, 2018). The influence of the astringency types on variations in the proanthocyanidins concentration is well-known in persimmon fruit (Akagi et al., 2009). However, little is known about the impact of the fruit astringency type on flavonoid metabolic pathway and its associated enzymes, genes, and TFs in persimmon fruit.

This study conducted transcriptomic and quasi-targeted metabolomic analyses to elucidate both gene expression and metabolite accumulated profiles in different stages of PCNA and PCA persimmon fruit. Specifically, dynamic changes in the expression of genes and TFs in flavonoid biosynthesis and the accumulation of a set of flavonoids were analyzed to clarify and compare the secondary metabolism of the persimmon fruit and its complex effects on the astringency and flavor components between PCNA and PCA persimmons.

## 2 Material and methods

### 2.1 Plant materials

Well-cultivated PCNA (‘Jiro’ and ‘Youhou’) and PCA (‘Zhongshi No.5’ and ‘Huojing’) persimmons were planted in the forest planting base of the Research Institute of Non-timber Forestry (34°55′18″–34°56′27″N, 113°46′14″–113°47′35″E), Yuanyang County, Henan Province, China. The fruits of the ‘Jiro’ and ‘Huojing’ persimmon fruit were sampled at the young-fruit stage (when the fruit had reached about 40% of the final size, stage 1), the fruit expansion stage (when the fruit had reached approximately 70% of its final size, stage 2), the turning stage (the initial change in the skin color of the fruit, stage 3) and the mature stage (fully developed fruit color without astringency, stage 4). Persimmon fruit from the ‘Zhongshi No.5’ and ‘Youhou’ varieties were also harvested at stage 4 and termed PCA1 and PCNA2, respectively. The four development stages (from stage 1 to stage 4) of the ‘Jiro’ and ‘Huojing’ fruit were labeled as Jiro_S1, Jiro_S2, Jiro_S3, Jiro_S4 and Huojing_S1, Huojing_S2, Huojing_S3, and Huojing_S4, respectively. Furthermore, fruits of 141 persimmon germplasms were also harvested at the mature stage (stage 4), and these samples were used for total flavonoids content detection. The fresh fruit was frozen immediately in liquid nitrogen and stored at -80° until used for RNA extraction and metabolic analyses.

### 2.2 Extraction and determination of total flavonoids

The total flavonoids of persimmon fruit were extracted and detected according to Han et al. (Han et al., 2021) with a few modifications. Briefly, fruit powder (precise weight 5 g) was extracted with 60% (v/v) ethanol in an ultrasonic bath (30 min). Total flavonoid content was determined by AlCl_3_-(HAc-NaAC) colorimetric method, and rutin with purity = 98% (Solarbio Science & Technology Co., Ltd.) were used as a standard. The absorbance was determined at 420 nm wavelength in a UV spectrophotometer. Total flavonoids content and astringency type of 141 persimmon germplasms are listed in **Supplementary Table 1**.

### 2.3 Metabolome data analysis process

The method used for metabolite identification was similar to that of Wang et al. (Wang et al., 2022). Samples of freeze-dried persimmon fruit (100 mg) were weighed into 1.0 mL of 70% aqueous methanol. Metabolite profiling was performed using an ExionLC™ AD system (SCIEX) coupled with a QTRAP^®^6500+ mass spectrometer (SCIEX) and equipped with Xselect HSS T3 column (2.1×150 mm, 2.5 μm) by Novogene Co., Ltd. (Beijing, China). The mobile phase included eluent A, consisting of 0.1% formic acid in water, and eluent B, consisting of 0.1% formic acid-acetonitrile. The analysis conditions were as follows: column temperature, 50˚C; injection volume, 1.5 μL; flow rate, 0.4 mL/min. The mobile phases were water. The gradient program of phase A/phase B was 98:2 (v/v) at 0 min, 98:2 (v/v) at 2 min, 0:100 (v/v) at 15 min, 0:100 (v/v) at 17 min, 98:2 (v/v), at 17.1 min and 98:2 (v/v) at 20 min. The qualitative analysis of metabolites was conducted according to the secondary spectral information using Novogene’s in-house database. Metabolite quantification was carried out using the triple quadrupole mass spectrometer’s multiple reaction monitoring (MRM) mode. The KEGG (Kyoto Encyclopedia of Genes and Genome) database (http://www.genome.jp/kegg/) (Kanehisa et al., 2004) and HMDB (Human Metabolome Database) database (http://www.hmdb.ca/) (Wishart et al., 2007) were used for metabolite annotation. The metabolites with *P*-value < 0.05 and fold change≥ 2 were considered as differentially accumulated flavonoids (DAFs).

### 2.4 Transcriptome data analysis

Total RNA was extracted using the TRIzol Total RNA Isolation Kit (Sangon, Shanghai, China), and a library was established. Bioanalyzer 2100 was used to assess the RNA integrity. The NovaSeq platform (Illumina, San Diego, CA, USA) sequencing generates 150 bp paired-end readings. HISAT2 software (Zhang et al., 2021) was used to map the filtered reads to the *D. kaki* reference genome (unpublished). The prediction of new transcripts was performed using StringTie (Shumate et al., 2022). FeatureCounts (Liao et al., 2014) was used to count the read numbers mapped to each gene. The FPKM of each gene was calculated according to the length of the gene and the read count mapped to the gene. DEseq2 (Love et al., 2014) was used to detect the differentially expressed genes (DEGs) between the two groups, with a |log2-fold change| ≥ 1 and padj ≤ 0.05. ClusterProfiler 4.0 (Wu et al., 2021) was used for DEGs in Gene Ontology (GO) (Ashburner et al., 2000) and KEGG (Kanehisa et al., 2004) functional enrichment analyses. Heatmaps and K-means clustering were prepared using the online software Hiplot (Li et al., 2022). The weighted gene coexpression network analysis (WGCNA) were constructed using all genes and were analyzed using WGCNA R package (Langfelder and Horvath, 2008). The networks were visualized using Cytoscape v3.9.1 (Shannon et al., 2003).

### 2.5 Quantitative RT-PCR analysis

The cDNA was synthesized from the high-quality total RNA using TRUE-script First-Strand cDNA Synthesis Kit (Kemix, Beijing, China). Reactions were performed with LightCycler 480 II (Roche), and PCR conditions were 95°C for 3 min, 45 cycles of 95°C for 5 s, and 55-60°C for 30 s. All analyses were conducted with three biological replicates. The relative expression of each sample was calculated by the 2−ΔΔCt method. The persimmon *GAPDH* gene was used as a reference gene (Du et al., 2019). All gene primers are listed in **Supplementary Table 2**.

## 3 Results

### 3.1 Correlation analysis between total flavonoids content and astringency type

The correlation between flavonoids content and fruit astringency were detected in the mature fruit of a natural population with different persimmon cultivars (**Supplementary Table 1**). The Pearson correlation coefficient (r) between total flavonoids content of persimmon fruit and astringency types of 141 persimmon resources was 0.415**, indicating a significant correlation between total flavonoids content and fruit astringency type (*P* < 0.01). The above information indicated that total flavonoids were differences between PCNA and PCA fruit.

### 3.2 Comparison of metabolites between mature PCNA and PCA persimmon fruit

To comprehensively define and compare the metabolite profiles between the PCNA and PCA persimmons, we evaluated metabolite compositions using a Quasi-Targeted metabolome. Mature fruits from the ‘Jiro’, ‘Youhou’, ‘Zhongshi No.5’, and ‘Huojing’ persimmon varieties were labeled as PCNA1, PCNA2, PCA1, and PCA2, respectively. Principal component analysis based on the data for all compounds separated all samples into four distinct groups, with each sample and its replicates forming a separate group, indicating that there were good correlations within group replicates and differences among the different groups (**Figure S1**). A total of 889 metabolites were identified in PCNA and PCA fruit, including five metabolite categories, namely, amino acids and their derivatives (170), flavonoids (135), carbohydrates and their derivatives (76), nucleotides and their derivates (65), and organic acid and its derivatives (62) (**Supplementary Table 3**).

**Figure 1:**
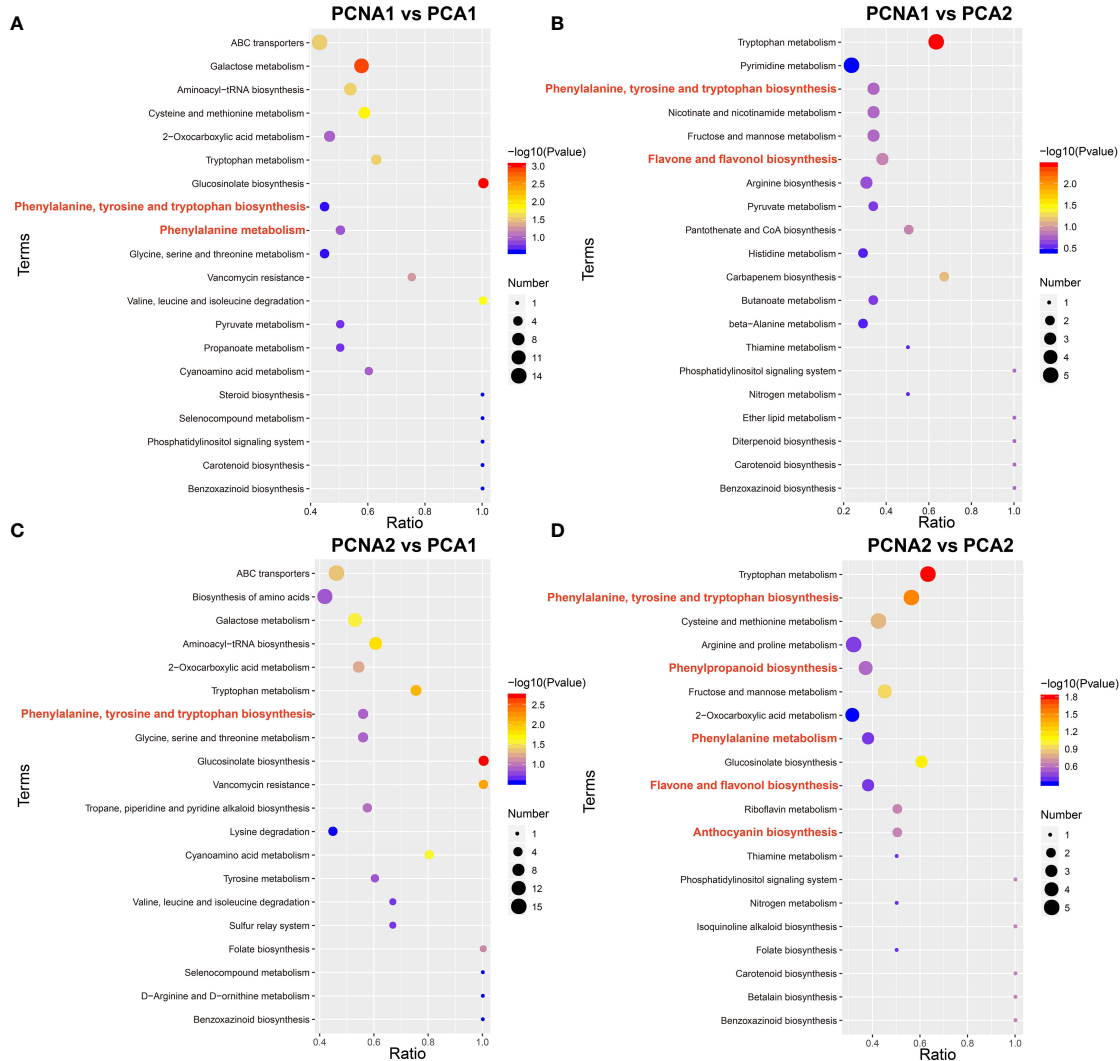
Comparison of metabolites between mature PCNA and PCA persimmon fruit. Scatter plot showing KEGG enrichment of DEGs between the PCNA and PCA groups. **(A)** PCNA1 vs. PCA1 group. **(B)** PCNA1 vs. PCA2 group. **(C)** PCNA2 vs. PCA1 group. **(D)** PCNA2 vs. PCA2 group.

To systematically identify and compare the metabolic pathways between the fruit of the PCNA and PCA genotypes, KEGG enrichment analysis was conducted on the differential metabolites of the four groups (PCNA1 vs. PCA1, PCNA1 vs. PCA2, PCNA2 vs. PCA1, and PCNA2 vs. PCA2). Phenylpropanoid biosynthesis (ko00940), phenylalanine metabolism (ko00360), flavonoid biosynthesis (ko00941), flavone and flavonol biosynthesis (ko00944), and anthocyanin biosynthesis (ko00942) were found to be significantly enriched. The KEGG annotation results suggested that flavonoid metabolism-related pathways were involved in the nutritional value and taste differences between PCNA and PCA fruit (**Figures 1A-D**). Given the importance of flavonoids to human health, the subsequent investigation were focused on flavonoid synthesis during PCNA and PCA fruit development.

### 3.3 RNA-Seq of PCNA and PCA persimmon developing fruits

To evaluate flavonoid variations between PCNA and PCA persimmons, the typical PCNA type ‘Jiro’ and PCA type ‘Huojing’ were selected for investigation. The fruit was harvested at four stages (S1–S4), namely, the young fruit stage (S1), expansion stage (S2), turning stage (S3), and mature stage (S4). After the removal of low-quality, poly-N, and adaptor sequences, the RNA-seq of the ‘Jiro’ and ‘Huojing’ fruit at the four stages yielded 160.80 GB of clean data. The filtered samples contained nearly 6.70 GB of high-quality data with an average Q30 base percentage of 92.42%. Approximately 85.43% of the reads mapped to the reference *D. kaki* genome, and 4416 novel genes were also identified. The transcriptome sequencing data were confirmed through qRT-PCR. Seven DEGs were randomly selected from the flavonoid metabolism pathway for qRT-PCR verification (**Figure S2**). The expression profiles of these genes were consistent with their FPKM values.

**Figure 2:**
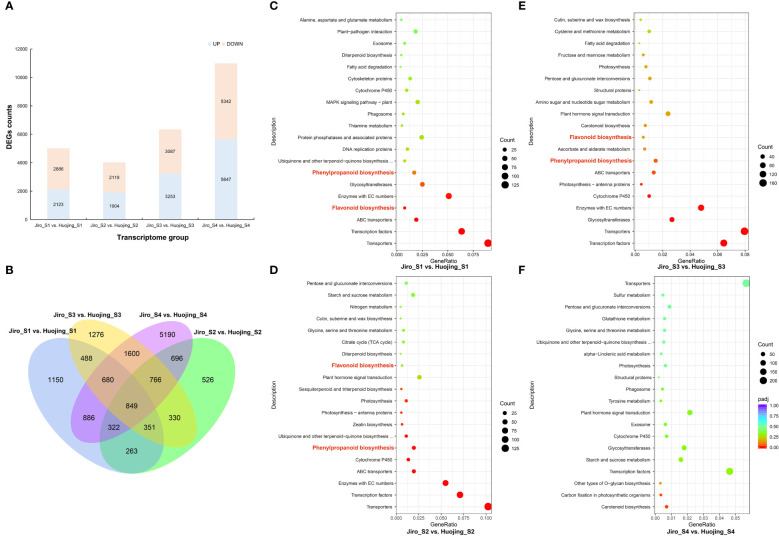
DEGs between ‘Jiro’ (PCNA type) and ‘Huojing’ (PCA type). **(A)** Summary of DEGs in different comparison groups of ‘Jiro’ and ‘Huojing’ fruit. **(B)** Venn diagram of DEGs. (c–f) Scatter plot showing KEGG enrichment of DEGs in four developmental stages in the ‘Jiro’ and ‘Huojing’ comparison groups. **(C)** Jiro_S1 vs. Huojing_S1 group. **(D)** Jiro_S2 vs. Huojing_S2 group. **(E)** Jiro_S3 vs. Huojing_S3 group. **(F)** Jiro_S4 vs. Huojing_S4 group.

The DEGs were compared using DESeq2 software, and selected DEGs were then analyzed. There were 5009, 4023, 6340, and 10 989 DEGs in the Jiro_S1 vs. Huojing_S1, Jiro_S2 vs. Huojing_S2, Jiro_S3 vs. Huojing_S3, and Jiro_S4 vs. Huojing_S4 groups, respectively (**Figure 2A**). Venn diagrams showed that 849 genes were differentially expressed in all the comparison groups, suggesting that these DEGs might perform critical functions in the regulation of flavonoids in PCNA and PCA fruit (**Figure 2B**). These DEGs were analyzed by the KEGG database to identify their associated pathways. This showed that the flavonoid biosynthesis (ko00941) and phenylpropanoid biosynthesis (ko00940) pathways were signicificantly enriched in the Jiro_S1 vs. Huojing_S1, Jiro_S2 vs. Huojing_S2, and Jiro_S3 vs. Huojing_S3 comparison groups. These results indicated that flavonoid biosynthesis-related pathways may play essential roles in different stages of PCNA and PCA fruit development (**Figures 2C-F**).

### 3.4 Differential gene analysis of flavonoid biosynthesis during the development of PCNA and PCA persimmon fruit

**Figure 3:**
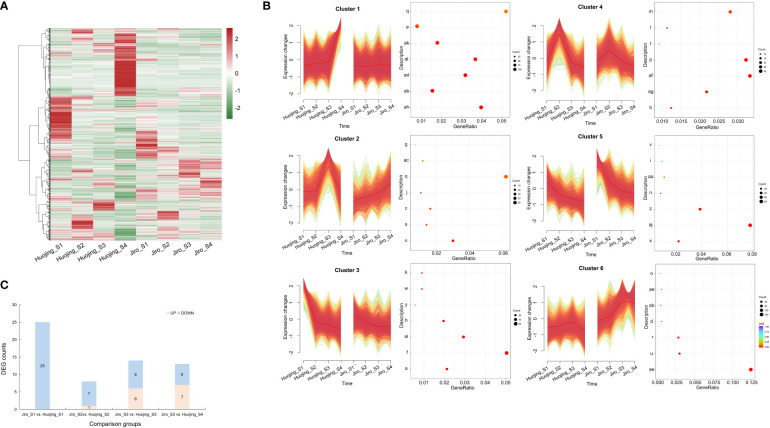
Expression patterns and KEGG analysis of genes in the transcriptomes of PCNA and PCA fruit. **(A)** Heatmap showing the overall common expression pattern. Heatmaps were constructed using the normalized gene expression values. **(B)** Expression profiles and KEGG annotations of six clusters. The y-axis of each cluster represents the KEGG categories, while the x-axis represents the rich factors. Red dots represent significantly overrepresented KEGG pathways. **(C)** Summary of flavonoid-associated DEGs in different comparison groups of ‘Jiro’ and ‘Huojing’. a, ABC transporters; b, alpha-Linolenic acid metabolism; c, Amino sugar and nucleotide sugar metabolism; d, Arachidonic acid metabolism; e, Autophagy- other; f, Chromosome and associated protein; g, Circadian rhythm-plant; h, Cytoskeleton proteins; i, Exosome; j, Fatty acid biosynthesis; k, Flavonoid biosynthesis; l, Glycine, serine and threonine metabolism; m, Glycosyltransferases; n, Ion channels; o, Lipopolysaccharide biosynthesis proteins; p, MAPK signaling pathway-plant; q, Membrane trafficking; r, Mitochondrial biogenesis; s, N-Glycan biosynthesis; t, Nitrogen metabolism; u, Oxidative phosphorylation; v, Phagosome; w, Phenylalanine, tyrosine and tryptophan biosynthesis; x, Photosynthesis; y, Photosynthesis-antenna proteins; z, Plant hormone signal transduction; aa, Porphyrin metabolism; ab, Promasome; ac, Protein export; ad, Protein processing in endoplasmic reticulum; ae, Ribosome; af, Ribosome biogenesis; ag, Ribosome biogenesis in eukaryotes; ah, Spliceosome; ai, Starch and sucrose metabolism; aj, Transcription factors; ak, Transcription machinery; al, Ubiquitin system.

Six expression patterns were generated through trend and clustering analyses of the DEGs in the ‘Jiro’ and ‘Huojing’ varieties of the four developmental stages, termed Cluster 1- Cluster 6 (**Figure 3A**). The genes in these expression profiles were functionally analyzed by KEGG annotation (**Figure 3B**). The expression levels of genes in Cluster 3 were higher in the ‘Huojing’ fruit than in the ‘Jiro’ fruit. Furthermore, the expression levels of genes in Cluster 3 decreased gradually with fruit maturation. The KEGG pathway analysis showed that genes in Cluster 3 were mainly involved in several primary metabolic processes, such as photosynthesis (ko00196) and starch and sucrose metabolism (ko00500), and secondary metabolisc processes, such as phenylalanine biosynthesis (ko00400) and flavonoid biosynthesis (ko00941). These results indicated that flavonoid metabolism was involved in the development of ‘Jiro’ and ‘Huojing’ fruit.

Genes involved in flavonoid biosynthesis were then selected from Cluster III based on the results of the KEGG analysis. A total of 44 DEGs involved in flavonoid biosynthesis were identified, including *CS*, *DAHPS*, *DHQS*, *DHD/SDH*, *EPSPS*, *PAL*, *C4H*, *4CL*, *CHS*, *CHI*, *F3H*, *DFR*, *ANS*, *FLS*, *OMT*, *SGT*, *UF3GT*, *LAC*, *ANR*, *AHA10* (ATPase), and *MATE* (**Supplementary Table 4**). The flavonoid-associated DEGs were more enriched in the Jiro_S1 vs. Huojing_S1 group than in the other groups, and most of the DEGs were decreased (**Figure 3C**). The analysis of the gene expression levels found that the numbers of DEGs gradually decreased during the developmental process and were specifically highly expressed in Huojing_S1. These results indicated that flavonoid metabolism occurred predominantly during in the early stage (S1) of fruit development and the expression of flavonoid biosynthesis genes in ‘Huojing’ was significantly higher than in the ‘Jiro’.

### 3.5 Construction of a flavonoid co-expression module during PCNA and PCA fruit development

To further identify the specific genes involved in regulating flavonoid metabolism during the development of PCNA and PCA persimmon fruit, 29 057 genes were used in a WGCNA analysis (**Figure S3**). To ensure high-scale independence (near 0.9), the β-value was set at 5 (**Figure S3A**). The adjacency and topological overlap matrices were then constructed (**Figures S3B, S3C**). Based on average hierarchical clustering and dynamic tree clipping, a total of 42 modules were obtained (**Figure S3D**). The expression levels of the MEbrown transcripts were found to be significantly higher in S1 compared with S2, and gene expression was significantly higher in Huojing_S1 than in Jiro_S1 (**Figure S3E**). The MEblue module contains 3502 genes and KEGG analysis of genes in these modules showed that the genes in the MEblue module were associated with flavonoid biosynthesis during the fruit development, specifically, phenylalanine biosynthesis (ko00400) and flavonoid biosynthesis (ko00941) (**Figure S3F**).

**Figure 4:**
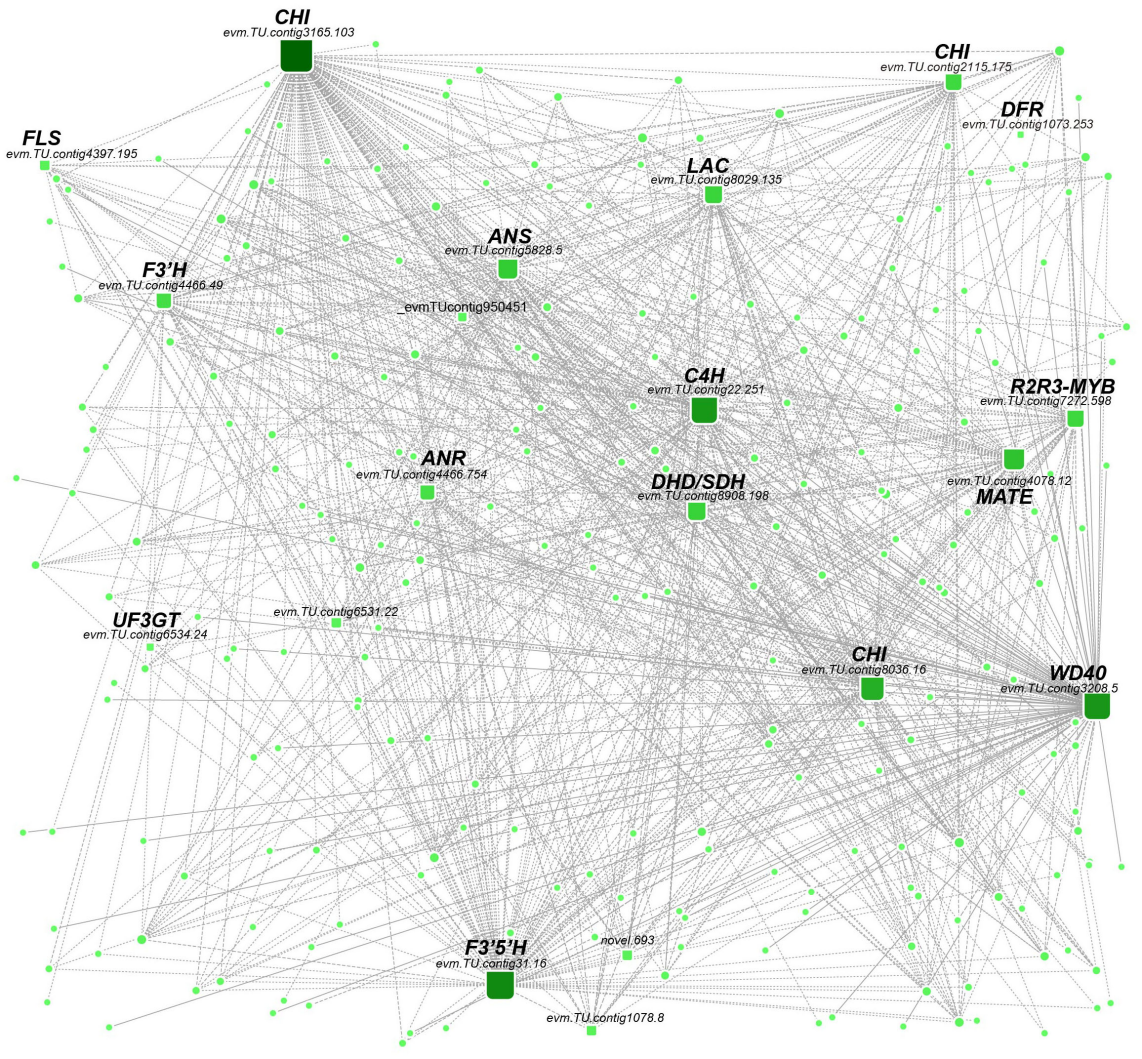
Weighted gene coexpression network analysis. The linkages between TFs and flavonoid-related structural genes in the MEblue module and the sizes of round rectangles were changed according to gene connectivity.

The genes with high connectivity in the MEblue module were further investigated as candidate key genes related to flavonoid metabolism. The top 10% of genes in terms of connectivity were selected as potential hub genes. Of these, 12 hubs were identified as potential regulators of flavonoid metabolism, including the upstream chorismic acid pathway gene *DHD/SDH* (*evm.TU.contig8908.198*); phenylpropanoid biosynthesis *PAL* gene (*evm.TU.contig9504.51*) and *C4H* gene (*evm.TU.contig22.251*); isoflavonoid biosynthesis genes *CHI* (*evm.TU.contig3165.103* and *evm.TU.contig8036.16*), and *CHS* (*evm.TU.contig2115.175*); flavone and flavanonols biosynthesis genes *F3H* (*evm.TU.contig4466.49*), *F3’5’H* (*evm.TU.contig31.16*), and *FLS* (*evm.TU.contig4397.195*); anthocyanidin biosynthesis genes *DFR* (*evm.TU.contig1073.253*), *ANR* (*evm.TU.contig4466.754*), *ANS* (*evm.TU.contig5828.5*), and *UF3GT* (*evm.TU.contig6534.24*); And flavonoid transport *MATE* (*evm.TU.contig4078.12*) gene. We observed the transcription factors *R2R3MYB* (*evm.TU.contig7272.598*) and *WD40* (*evm.TU.contig3208.5*) also showed higher connectivity and were closely associated with the above structural genes. Therefore, these TFs might participate in regulating the expression levels of the structural genes in flavonoid biosynthesis (**Figure 4**).

### 3.6 Analysis of flavonoid metabolites during the development of PCNA and PCA persimmon fruit

**Figure 5:**
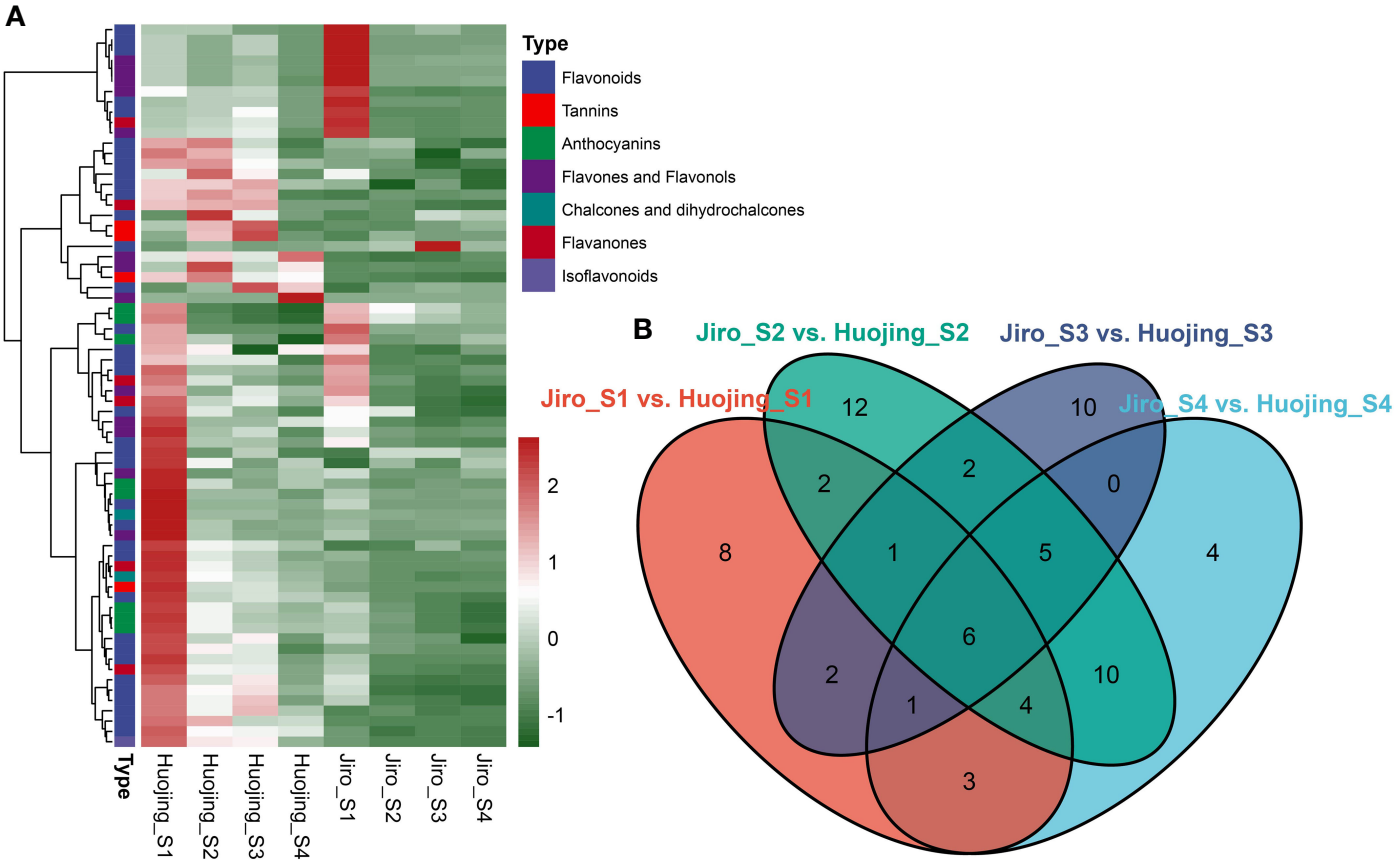
Differentially accumulated flavonoids (DAFs) in ‘Jiro’ (PCNA type) and ‘Huojing’ (PCA type). **(A)** Heatmap of the DAFs. The Heatmaps depict the normalized gene expression values, representing the mean value of three biological replicates. **(B)** Venn diagram of DAFs.

To further confirm flavonoid differences in the PCNA and PCA persimmon fruit during the developmental process, Quasi-Targeted metabolomic analysis of flavonoid compounds was used to evaluate the four developmental stages of PCNA and PCA persimmon fruit (**Figure 5**). In total, 135 flavonoids were identified in LS and JS fruit at different stages. These flavonoids included 75 flavonoids, 22 flavones and flavonols, 15 flavanones, 11 anthocyanins, four chalcones and dihydrochalcones, four isoflavonoids, and four tannins (**Supplementary Table 5**).

A total of 70 DAFs were identified among all the comparison groups, including 36 flavonoids, 13 flavones and flavonols, eight anthocyanins, six flavanones, four tannins, two chalcones, and dihydrochalcones, and one isoflavonoid, of which 28 flavonoids were glycosides. There were 27 DAFs in Jiro_S1 vs. Huojing_S1, 33 DAFs in Jiro_S1 vs. Huojing_S1, 41 DAFs in Jiro_S1 vs. Huojing_S1, and 27 DAFs in Jiro_S1 vs. Huojing_S1. Most of the DAFs were significantly higher in Huojing_S1 than in Jiro_S1, with only Procyanidin B2 and Procyanidin B3 were upregulated in at least two stages. The DAFs in the ‘Huojing’ variety were stronger than in the ‘Jiro’, which was consistent with the results of the RNA-seq analysis. Further analysis revealed that 8, 4, 12, and 10 flavonoid metabolites were stage-specific for S1, S2, S3, and S4, respectively (**Figures 5A, B**). In addition, six metabolites were differentially expressed at all developmental stages, including 3,7-dimethoxykaempferol-C-glucoside, corilagin, gallic acid, laricitrin, methyl gallate, and phlorizin. These results further confirmed that there were significant differences in the flavonoids between PCNA and PCA persimmons, and that flavonoid biosynthesis pathways play an essential role in PCNA and PCA fruit development.

### 3.7 Analysis of flavonoid biosynthesic genes and metabolies during the development of PCNA and PCA persimmon fruit

**Figure 6:**
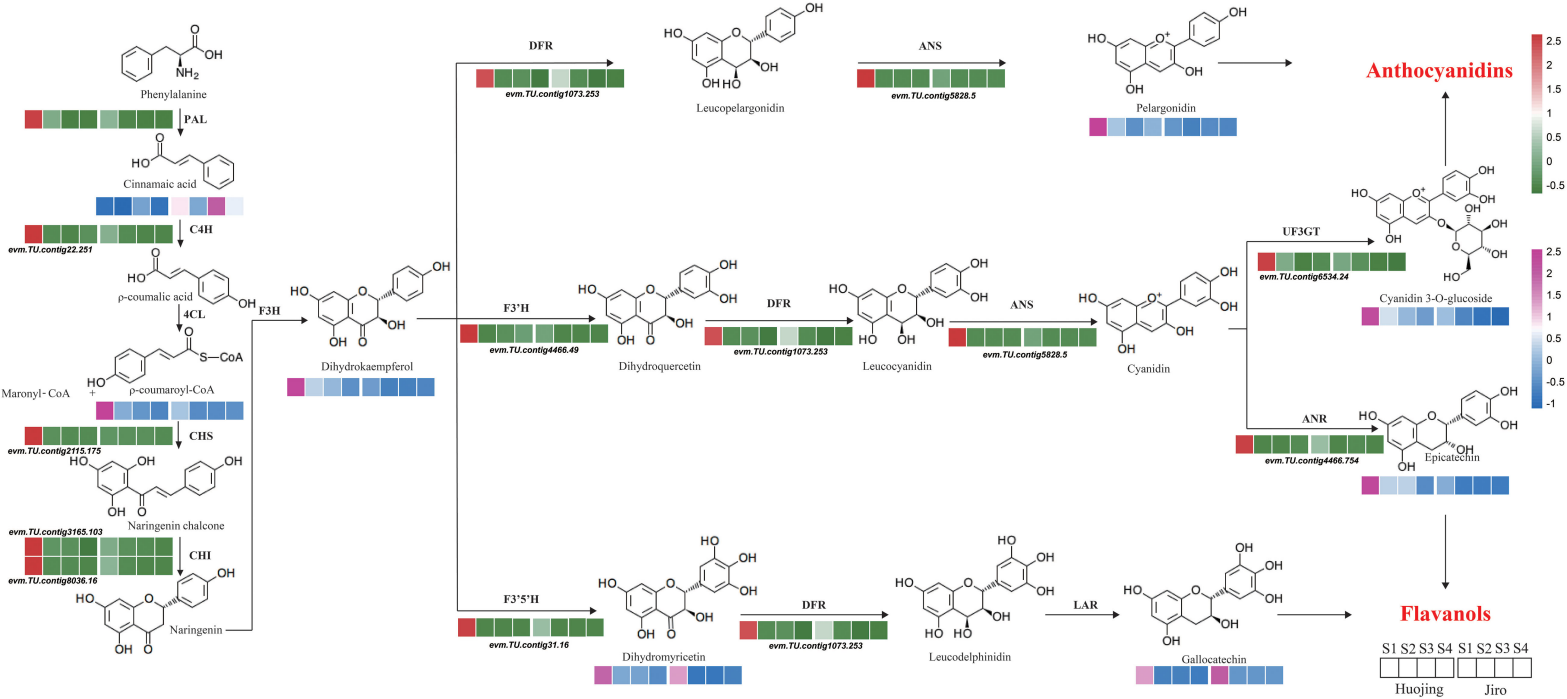
Diagram of phenylpropanoid and flavonoid biosynthetic pathways involving DEGs and DEMs. Heatmaps represent the normalized expression values.

Based on the KEGG enrichment and WGCNA analyses, a flavonoid biosynthetic pathway was systematically constructed showing the expression levels of structural genes and the flavonoid contents during PCNA and PCA persimmon fruit development (**Figure 6**). Eleven structural genes and eight flavonoids were mapped to the pathway. Two structural genes (*PAL* and *C4H*) participated in the upstream phenylpropanoid pathway and nine structural genes (*CHS*, 2 *CHI*s, *F3’H*, *F3’5’H*, *DFR*, *ANS*, *ANR*, and *UF3GT*) participated in the flavonoid biosynthetic pathway. *PAL2* catalyzes the transformation of phenylalanine to cinnamic acid and as the expression of *C4H* was significantly higher in Huojing_S1 than in Jiro_S1, there was a upregulation of ρ-coumaroyl-CoA, with downregulation of cinnamic acid. Subsequently, a series of flavonoid structural genes, *CHS*, *CHI*s, *F3’5’H*, and *F3’H* showed significantly higher expression levels in Huojing_S1. Thus, some crucial intermediates such as dihydrokaempferol and dihydromyricetin, produced by the enzymes encoded by these structural genes, accumulated highly in Huojing_S1 than in Jiro_S1. All genes related to anthocyanin biosynthesis, such as *F3’5’H*, *DFR*, *ANS*, *ANR*, and *UF3GT*, were significantly downregulated in ‘Jiro’, resulting in lower accumulation of anthocyanins, such as pelargonidin chloride and cyanidin 3-O-glucoside in ‘Jiro’ than ‘Huojing’, which might be associated with the differences in flesh color between ‘Jiro’ and ‘Huojing’. However, the levels of the colorless metabolites catechin (gallocatechin) and proanthocyanidin (procyanidin B2 and procyanidin B3) metabolites were significantly higher in Jiro_S4 than Huojing_S4. These findinge demonstrated the main contributions of these eight DAFs to the differences in flavonoid biosynthesis between PCNA (‘Jiro’) and PCA (‘Huojing’), and the critical regulatory roles of 11 genes associated with flavonoid synthesis were hypothesized.

## 4 Discussion

As a fruit tree cultivated worldwide, the persimmon has essential ecological, economic, and social value. Presently, persimmon trees are mainly grown for their fruit, which can be eaten fresh or dried. Persimmon fruit varies greatly in terms of specific qualities, with significant variations observed between PCNA and non-PCNA persimmons (Novillo et al., 2016; Yildiz and Kaplankiran, 2018). Volatile compounds also vary significantly between PCNA and PCA fruit, especially in terms of aldehydes (Elhadi, 2017). In addition to soluble tannin, the astringency type also affected the contents of total phenolics, flavonoids, soluble solids, individual sugars, as well as antioxidant capacity (Novillo et al., 2016; Yildiz and Kaplankiran, 2018). These studies provide a preliminary survey of how the astringency type affects the quality of the persimmon fruit; however, given the importance of flavonoids, the flavonoid composition and potential mechanisms involved in the regulation of flavonoid biosynthesis between the PCNA and PCA fruit varieties still require clarification.

With the rapid development of transcriptome sequencing, many studies have attempted to elucidate the molecular basis of flavonoid biosynthesis *via* RNA-seq. The key genes involved in flavonoid biosynthesis were identified by stage-specific transcriptomic analysis in the petals of *Camellia nitidissima* (Liu et al., 2023). Changes in the key genes and flavonoid metabolites were also investigated using metabolomics and transcriptomics in the developing exocarp and embryo of hickory (Chen et al., 2022). Metabolomics examines the overall metabolic profile of plant samples through high-throughput detection and data processing (Foito and Stewart, 2018) and can thus provide a reliable method for investigating compounds contributing to the flavor of the persimmon fruit. GC-MS has been used previously to identify volatile components in persimmon fruit (Besada et al., 2013; Elhadi, 2017). Nineteen polyhydroxyphenols were found to be reduced in CO2-treated fruit using untargeted metabolomics analysis, suggesting that persimmon browning might be caused by phenolic compounds (Han et al., 2022). Differences in metabolites between five Japanese persimmons were investigated using NMR (Ryu et al., 2019). In this study, a comprehensive transcriptomic and metabolite analysis was conducted to determine the differences in flavonoid composition between PCNA and PCA persimmons and identify genes related to flavonoid biosynthesis.

Flavonoid biosynthesis is complicated and diverse and requires the substrates derived from the phenylpropanoid pathway (Liu et al., 2021). Phenylpropanoid biosynthesis, flavonoid biosynthesis, flavone and flavonol biosynthesis, phenylalanine metabolism, isoflavone biosynthesis, and anthocyanin biosynthesis were observed to be enriched during the development of hickory fruit (Chen et al., 2022). In this study, a full-spectrum metabolomic determination of persimmon fruit was performed using liquid chromatography and triple quadrupole mass spectrometry in the MRM mode. This resulted in the identification of a total of 135 flavonoids, greatly broadening our knowledge of flavonoids in ripening persimmon fruit. KEGG analysis of the differential accumulated metabolites in PCNA and PCA persimmons showed that pathways involved in phenylpropanoid biosynthesis, flavonoid biosynthesis, phenylalanine metabolism, anthocyanin biosynthesis, and flavone and flavonol biosynthesis were significantly enriched, indicating not only the high accumulation of flavonoids in persimmons but also that the flavonoids differed significantly between PCNA and PCA persimmons, which may be responsible for the differences in nutritional value and taste between the PCNA and PCA fruit.

In this study, a total of 70 differential accumulated flavonoids were identified between the fruit of the PCNA (‘Jiro’) and PCA (‘Huojing’) persimmon varieties at different developmental stages, of which the top three were flavonoids (36), flavones and flavonols (13), and anthocyanins (8). Twenty-eight DAFs were glycosides, which were mainly glycosylated derivatives of quercetin and cyanidin. Quercetin is mostly present in plants as glycosides and has been reported in foods such as onions, apples, broccoli, and tea, and it also has beneficial effects on health (Zheng et al., 2017). Glycoside modifications enhance the water solubility, structural complexity, and molecular stability of flavonoids (Bai et al., 2022). Thus, flavonoids with glycosides could play essential roles in plant growth, hormonal balance, and the elimination of toxic endogenous and exogenous substances (Wang et al., 2017; Bai et al., 2022); it has, for instance, been shown that delphinidin and its glycosides enhance plant resistance to a wide range of biotic and abiotic stresses (Silva et al., 2017).

In the early stages of flavonoid biosynthesis, the PCNA (‘Jiro’) and PCA (‘Huojing’) cultivars differed in the expression of the *C4H* (*evm.TU.contig22.251*), *PAL* (*evm.TU.contig9504.51*), *CHI* (*evm.TU.contig3165.103 and evm.TU.contig8036.16*), *and CHS* (*evm.TU.contig2115.175*) genes. L-phenylalanine is converted to cinnamic acid by *PAL*, the frist enzyme in the flavonoid biosynthetic pathway (Heldt and Piechulla, 2011). In addition to *PAL*, *C4H*, *CHS*, and *4CL* play critical roles in the synthesis of crucial secondary metabolites such as lignin, phenolic acids, coumarin, flavonoids, and anthocyanins (Chen et al., 2022; Xia et al., 2022). Subsequently, ρ-Coumaric-CoA produces naringenin, which is catalyzed by *CHS* and *CHI* (Yuan et al., 2022). *CHS* is a key initiating enzyme and forms part of a multi-gene family in most species (Niesbach-Klösgen et al., 1987). *CHS* (*evm.TU.contig2115.175*) expression was observed to be significantly lower in cultivar ‘Jiro’ compared with ‘Huojing’ at the early stage. Two DEGs that encode *CHI* (*evm.TU.contig3165.103* and *evm.TU.contig8036.16*) were also identified between PCNA and PCA persimmons; these two *CHI* genes were highly expressed in cultivar ‘Huojing’ at stage 1. The expression levels of these genes might influence flavonoid metabolism in PCNA and PCA persimmons.

At the late stage of flavonoid biosynthesis, the *F3H* (*evm.TU.contig4466.49*), *F3’5’H* (*evm.TU.contig31.16*), and *FLS* (*evm.TU.contig4397.195*), *DFR* (*evm.TU.contig1073.253*), *ANS* (*evm.TU.contig5828.5*), *ANR* (*evm.TU.contig4466.754*), and *UF3GT* (*evm.TU.contig6534.24*) genes showed differential expression between PCNA (‘Jiro’) and PCA (‘Huojing’) persimmon fruit. Two key enzymes, *F3’H* and *F3’5’H*, regulate the hydroxylation of naringenin and dihydrokaempferol at the 3’ position or both the 3’ and 5’ locations in the B ring (Bailey et al., 2003), and the products are crucial intermediates in the biosynthesis of anthocyanins and proanthocyanidins (Jeong et al., 2006). Thus, the genes encoding the *F3’H* and *F3’5’H* enzymes have been extensively studied in horticultural plants such as cyclamen (Boase et al., 2010), tea (Guo et al., 2019) and grapes (Jeong et al., 2006). In this study, *F3’H* (*evm.TU.contig4466.49*) showed significantly lower expression in cultivar ‘Jiro’ compared with ‘Huojing’ at the early stage when it catalyzes dihydrokaempferol to produce dihydroquercetin, a substrate of cyanidin. *F3’5’H* (*evm.TU.contig31.16*) catalyzes the synthesis of dihydromyricetin, a substrate of leucodelphinidin. These results are consistent with those of similar studies of *Rhododendron pulchrums* (Xia et al., 2022).

*LAR* and *ANS* play essential roles in the synthesis of proanthocyanins and anthocyanins; both are downstream genes of the flavonoid biosynthetic pathway and catalyze leucocyanidin into catechins and cyanidins, respectively (Springob et al., 2003). *ANR* and *LAR* encode key enzymes involved in the production of 2,3-cis-flavan-3-ols [(-)-epigallocatechin (ECG), (-)-epicatechin (EC), and (-)-epi-gallocatechin 3-O-gallate (EGCG)] and 2,3-trans-flavan-3-ols [(+)-gallocatechin (GC) and (+)-catechin (CA)] respectively (Ikegami et al., 2007). Interestingly, there were no differences in the *LAR* expression level between the two cultivars but *ANR* (*evm.TU.contig4466.754*) was expressed at higher levels in cultivar ‘Huojing’, a finding similar to previous studies showing that the expression level of *DkANR* was much higher than that of *DkLAR* during proanthocyanin accumulation (Akagi et al., 2009). This resulted in a lower epicatechin content in the PCNA persimmon and was one of the main reasons for the reduction in the proanthocyanin contents in PCNA types. Cyanidin and pelargonidin usually provide the red pigment in fruit and flowers (Harborne and Williams, 2000). In this study, *UF3GT* (*evm.TU.contig6534.24*) catalyzed the formation of cyanidin 3-O-glucoside, and *ANS* (*evm.TU.contig5828.5*) also catalyzed naringin to produce pelargonidin chloride; both two genes were expressed at higher levels in ‘Huojing’ than in ‘Jiro’, which might result in the accumulation of less red pigmentation in PCNA persimmons.

Genes involved in flavonoid biosynthesis are mainly regulated by the *MYB*, *bHLH*, and *WD40* TFs and their *MBW* complex in plants (Hichri et al., 2011), such as rose (Shen et al., 2019) and pears (Premathilake et al., 2020). *R2R3MYB* TFs are core members of the *MBW* complex and are involved in the regulation of flavonoid biosynthesis through binding to the promoter regions of structural genes (Yoshida et al., 2015). In pears, *PpMYB17* has been shown to positively regulate flavonoid biosynthesis by activating the structural genes *PpCHS*, *PpCHI*, *PpF3H*, and *PpFLS* in fruit (Premathilake et al., 2020). In persimmon, the combined action of *DkMYB2*, *DkMYC1*, and *DkMYB4* (*MBW*) increases the expression levels of the *ANR* gene involved in the biosynthesis of the proanthocyanin precursor cis-flavan-3-ols (Gil-Muñoz et al., 2020), supporting the above findings on the structural gene *ANR* and the cis-flavan-3-ols epicatechin content. Besides, *DkMYB14* in the Chinese PCNA (C-PCNA) persimmon was found to suppress proanthocyanin biosynthesis and activate acetaldehyde biosynthesis, resulting in the deastringency of the C-PCNA persimmon fruit (Chen et al., 2021). *MYB82* is involved in trichome development (Liang et al., 2014) and has potential roles in anthocyanin biosynthesis in *Arabidopsis* (Yang et al., 2013). In this study, an *R2R3MYB* (*evm.TU.contig7272.598*) was identified by WGCNA, which was homologous to *AtMYB82* and *BrMYB82* (Yang et al., 2013), indicating a potential role of *MYB82* in anthocyanin biosynthesis regulation; however, the mechanism remains requires further investigation and confirmation.

In conclusion, a comprehensive metabolomic and transcriptomic analysis of PCNA (‘Jiro’) and PCA (‘Huojing’) persimmon fruit was conducted. The concentration of flavonoids in ‘Huojing’ was found to be significantly higher than in ‘Jiro’ fruit, especially the concentrations of the proanthocyanin precursor 2,3-cis-flavan-3-ols epicatechin and anthocyanin cyanidin derivatives. Combined WGCNA and KEGG analyses showed that genes such as *PAL*, *C4H*, *CHI*, *CHS*, *F3H*, *F3’5’H*, *FLS*, *DFR*, *ANR*, *ANS*, and *UF3GT* involved in the phenylpropanoid and flavonoid biosynthetic pathways might be the major factors impacting the proanthocyanin precursor flavan-3-ols and the anthocyanin content. Moreover, the *R2R3MYB* (*evm.TU.contig7272.598*) and *WD40* (*evm.TU.contig3208.5*) TFs showed significant connections with the above structural genes. This study provides basic information on flavonoid biosynthesis and regulatory network in persimmon fruit and lays a foundation for ongoing investigations on the influence of astringency types on flavor components in PCNA and PCA persimmon.

## Data availability statement

The datasets presented in this study can be found in online repositories. The names of the repository/repositories and accession number(s) can be found below: https://www.ncbi.nlm.nih.gov/, https://www.ncbi.nlm.nih.gov/bioproject/PRJNA910302.

## Author contributions

YW and YS carried out the experiments and wrote this manuscript. YW and YS participated in the data analysis. HL, ZW, SD, and PS participated in the sampling and investigation; WH and JF conceived and designed the experiments and helped draft and review the manuscript. All authors contributed to the article and approved the submitted version. This article complies with ethical standards.

## Supplementary material

Supplementary Figure 1 Principal component analysis score plot of all metabolites in mature PCNA and PCA persimmon fruit.

Supplementary Figure 2 Verification of transcriptomic data by qRT-PCR analysis of the expression of seven genes.

Supplementary Figure 3 WGCNA analysis. **(A)** Soft threshold selection; **(B)** Module hierarchical clustering tree; **(C)** The gene co-expression module; **(D)** The correlation between samples and modules; **(E)** Gene expression pattern of the MEblue module; and **(F)** KEGG enrichment analysis of the MEblue module.
