# Supplementary figures and images for: Comparative transcriptomic and metabolomic analyses reveal differences in flavonoid biosynthesis between PCNA and PCA persimmon fruit
Source: Front Plant Sci. 2023 Feb 27;14:1130047. doi: 10.3389/fpls.2023.1130047 (PMC10009267; doi:10.3389/fpls.2023.1130047)

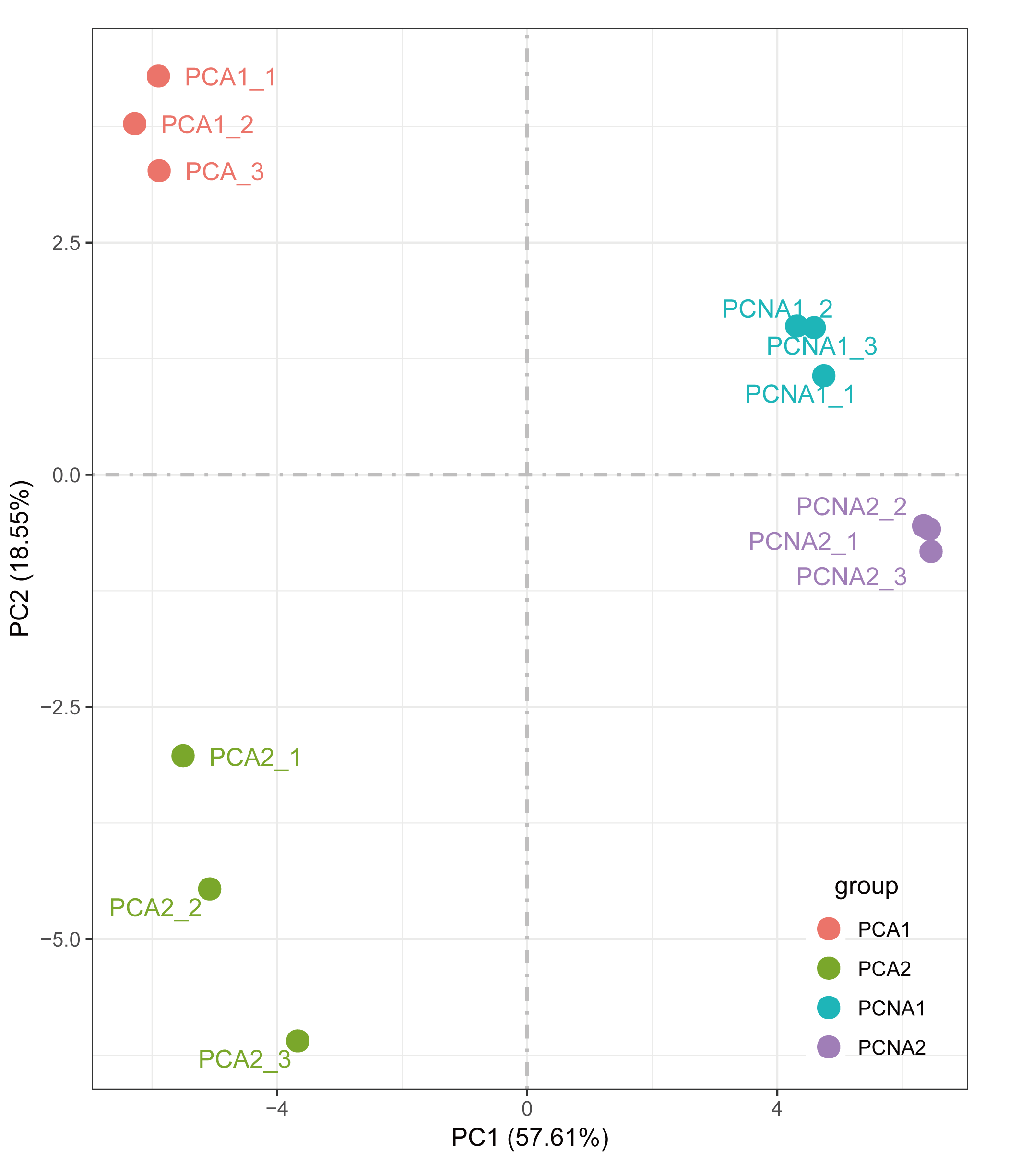

Supplement: Supplementary Figure 1 — Principal component analysis score plot of all metabolites in mature PCNA and PCA persimmon fruit. [file Image_1.png]
